# Total cerebrospinal fluid IgA as a candidate adjunctive biomarker associated with autoimmune encephalitis: a retrospective case–control study

**DOI:** 10.3389/fneur.2026.1934933

**Published:** 2026-08-27

**Authors:** Dongxing Wang, Ling Tan, Pengzhu Yang, Bo Lv, Yu Wang, Jiaping Xu, Yanlin Zhang, Yongjun Cao

**Affiliations:** 1 Department of Neurology, The Second Affiliated Hospital of Soochow University, Suzhou, China; 2 Department of Pharmacy, The Second Affiliated Hospital of Soochow University, Suzhou, China; 3 Information and Big Data Center, The Second Affiliated Hospital of Soochow University, Suzhou, China

**Keywords:** autoimmune encephalitis, biomarkers, cerebrospinal fluid, early diagnosis, immunoglobulin A

## Abstract

**Background:**

Autoimmune encephalitis (AE) is a rare but severe immune-mediated disease of the central nervous system. Early diagnosis remains challenging, and total cerebrospinal fluid (CSF) IgA is a routinely measurable parameter. However, its association with AE and its potential value as a candidate adjunctive biomarker remain unclear.

**Objective:**

To investigate whether total CSF IgA is independently associated with AE and to explore its potential as a candidate adjunctive biomarker for identifying AE cases.

**Methods:**

We conducted a single-center retrospective case–control study at the Second Affiliated Hospital of Soochow University (January 2010–June 2025), enrolling patients with autoimmune encephalitis and symptomatic controls with non-inflammatory, non-organic neurological disorders. Clinical, laboratory, and neuroimaging data were collected. Logistic regression was used to assess the independent association of total CSF IgA with autoimmune encephalitis and to construct a prediction model. Model performance was evaluated using receiver operating characteristic curves, calibration plots, decision curve analysis, and bootstrap internal validation.

**Results:**

A total of 101 patients with AE and 80 controls were included. Total CSF IgA levels were significantly higher in patients with AE than in controls (median 6.03 vs. 1.46 mg/L, *p* < 0.001). In the initial multivariable logistic regression model, total CSF IgA was independently associated with AE (OR = 31.63, 95% CI: 2.13–469.09, *p* = 0.011). The final model incorporated CSF IgA, CSF WBC count, CSF total protein, CSF albumin, blood WBC count, and serum globulin, with CSF IgA remaining independently associated with AE (adjusted OR, 20.53; 95% CI, 4.77–167.45). Although the wide confidence interval warranted caution in interpreting the magnitude of the association. The overall model showed excellent discrimination (AUC = 0.990, 95% CI: 0.982–0.999), good calibration (Brier score, 0.037), and a clear net clinical benefit on decision curve analysis.

**Conclusion:**

Total CSF IgA was independently associated with AE, and a multivariable model combining CSF IgA with routine laboratory parameters showed excellent discrimination and good calibration in distinguishing AE from symptomatic controls. As a rapidly measurable parameter, it shows promise as a candidate adjunctive biomarker. However, its specificity in distinguishing AE from clinical mimics remains uncertain, warranting validation in larger, independent studies.

## 1 Background

Autoimmune encephalitis (AE) is a rare but potentially life-threatening neuroinflammatory disorder mediated by autoantibodies targeting neural antigens. Because delayed diagnosis and treatment may lead to severe neurological sequelae or death, AE is regarded as a neurological emergency requiring prompt recognition and timely initiation of immunotherapy (1, 2).

Although the 2016 international consensus criteria proposed by Graus et al. (3) have facilitated early diagnosis before antibody results are available, the clinical diagnosis of AE remains challenging. Misdiagnosis may occur because of non-specific neuropsychiatric presentations, overinterpretation of serum antibody positivity, and inappropriate application of criteria for antibody-negative cases (4–6). Moreover, Comprehensive testing for anti-neural antibodies is not uniformly available across clinical settings and may require a long turnaround time (7).

Immunoglobulin A (IgA) is classically recognized as the principal effector immunoglobulin of mucosal immunity. However, emerging evidence suggests that IgA-related immune responses may also participate in systemic and central nervous system (CNS) immune regulation (8–10). Gut-derived IgA-positive plasma cells have been detected in the CNS in experimental autoimmune encephalomyelitis and multiple sclerosis, supporting a potential role for IgA in neuroinflammation (11, 12). In addition, IgA-mediated neutrophil activation has been implicated in severe dengue, and MOG-IgA has shown potential diagnostic relevance in CNS demyelinating disorders. Together, these findings suggest that IgA may contribute to neuroinflammatory and CNS immune-mediated diseases (13, 14).

Nevertheless, previous studies have mainly focused on antigen-specific IgA antibodies, whereas the diagnostic relevance of total cerebrospinal fluid (CSF) IgA, a routinely available parameter in clinical laboratory testing, remains unexplored in AE. To address this gap, we conducted a retrospective case–control study to evaluate whether total CSF IgA could serve as a potentially useful adjunctive biomarker for early identification of AE.

## 2 Methods

### 2.1 Study design and participants

This single-center, retrospective case–control study adhered to a pre-defined protocol for participant selection to minimize bias. Candidate cases for the autoimmune encephalitis group were independently screened by two experienced physicians. A parallel, blinded screening process was implemented for the symptomatic control group by two deputy chief physicians unaware of the study grouping. For both groups, concordant selections were included, and any discordant cases underwent blinded adjudication by a third senior physician, with a final consensus reached by an expert panel. All statistical analyses were conducted by a PhD statistician with over 5 years of clinical research experience, according to a pre-specified analysis plan.

### 2.2 Study subjects

#### 2.2.1 Autoimmune encephalitis

Inclusion criteria (Patients must meet all of the following):

1. Compliance with internationally recognized diagnostic criteria for AE (3):
  1. Subacute onset (rapid progression within < 3 months) with at least one of the following core symptoms:
    - Working memory deficits (short-term memory loss);
    - Altered mental status, including lethargy, impaired consciousness, or personality change;
    - Psychiatric manifestations;
  2. And at least one of the following objective supportive evidence:
    - New focal central nervous system findings;
    - Seizures not explained by a previously known seizure disorder;
    - CSF pleocytosis (> 5 × 10^6^/L). In cases with significant CSF red blood cell contamination (e.g., from traumatic tap), the following correction formula was applied: True CSF WBC = actual CSF WBC – [(Blood WBC × CSF RBC) / Blood RBC];
    - MRI features suggestive of encephalitis
2. Age: Between 18 and 80 years, inclusive.
3. Symptom onset to lumbar puncture: The interval from symptom onset to lumbar puncture was ≤ 2 weeks.
4. CSF neural antibody testing: Positive for neural autoantibodies.
5. Data completeness: Availability of complete clinical data, including CSF analysis (pressure, routine biochemistry, and immunoglobulins IgA, IgG, IgM), serum and CSF antibodies, electroencephalography (EEG), neuroimaging, and comprehensive blood tests (complete blood count, liver and renal function tests, electrolytes, glucose, thyroid function, viral serology, autoimmune antibodies, tumor markers, and C-reactive protein).

Exclusion criteria (patients were excluded if they met any of the following criteria):

1. CNS infections and infection-related conditions
  1. Confirmed or suspected CNS infection, including viral (e.g., HSV, VZV, HHV-4, HHV-6, enteroviruses), bacterial, fungal, tuberculosis, spirochetal (*T. pallidum*), and parasitic agents.
  2. Other active systemic infections (e.g., pneumonia, urinary tract infection, gastrointestinal infection) occurring within 4 weeks prior to enrollment.
  3. History of hepatitis virus (HAV, HBV, HCV, HDV, HEV), HIV, or syphilis infection.
2. Other CNS disorders
  1. Coexisting or previously diagnosed other CNS immune-mediated or inflammatory disorders, including multiple sclerosis, neuromyelitis optica spectrum disorders, CNS vasculitis, and neurosarcoidosis.
  2. Primary CNS tumors, cerebrovascular diseases, neurodegenerative diseases or traumatic brain injury.
  3. Metabolic or toxic encephalopathies.
3. Systemic autoimmune diseases and immunodeficiency states:
  1. Pre-existing or active systemic autoimmune diseases (e.g., systemic lupus erythematosus, rheumatoid arthritis, Sjögren’s syndrome).
  2. Receipt of immunosuppressive therapy (e.g., corticosteroids continuously for >1 week, chemotherapy, or biologic agents) within 3 months prior to enrollment.
  3. History of organ transplantation.
4. Hepatic or renal dysfunction: Alanine aminotransferase or aspartate aminotransferase >2 × upper limit of normal (ULN), or total bilirubin >1.5 × ULN, or presence of decompensated cirrhosis; Estimated glomerular filtration rate (eGFR) < 90 mL/min/1.73 m^2^, or serum creatinine above the ULN.
5. Diabetes mellitus, thyroid disease, Guillain–Barré syndrome, or hematological malignancies.
6. Substance use: History of chronic alcohol dependence or drug abuse/addiction.
7. Other conditions: Comorbid chronic nasal, oral, or skin diseases; pregnancy or lactation.

#### 2.2.2 Symptomatic control group

Due to ethical constraints prohibiting the acquisition of CSF from healthy volunteers, the symptomatic control group was selected from the medical records database of the Second Affiliated Hospital of Soochow University. These were patients who underwent lumbar puncture for non-specific neurological symptoms (e.g., dizziness, neuralgia, or insomnia) but were ultimately diagnosed with non-inflammatory, non-organic conditions.

Inclusion criteria (controls must meet all of the following):

1. Clinical presentation: Underwent lumbar puncture for symptoms such as dizziness, neuralgia, or insomnia.
2. Age: Between 18 and 80 years, inclusive.
3. Final diagnosis: Discharge diagnosis belonging to the following non-inflammatory, non-organic disorders: tension-type headache, primary insomnia, benign paroxysmal positional vertigo, or peripheral vestibular vertigo.
4. Normal CSF parameters:
  - Pressure: 80–180 mmH₂O.
  - WBC count: ≤5 × 10^6^/L.
  - RBC count: 0.
  - Total protein: 150–450 mg/L.
  - Albumin: 0–350 mg/L.
  - Normal glucose: 2.5–4.5 mmol/L.
  - CSF Chloride: 120–132 mmol/L.
  - Negative fungal smear and culture.
5. Normal blood test results:
  - Normal liver and kidney function (defined as having ALT, AST, total bilirubin, and albumin levels within the institutional reference range, and an estimated glomerular filtration rate ≥90 mL/min/1.73m^2^).
  - Normal fasting blood glucose, serum glucose level <6.1 mmol/L.
  - Normal electrolytes and thyroid function (defined as serum sodium, potassium, chloride, calcium, TSH, FT3 and FT4 levels all within the normal reference ranges of our institutional laboratory).
  - Negative tumor markers, viral serology (HAV, HBV, HCV, HDV, HEV, HIV, syphilis), and autoantibodies.
  - WBC count: 3.5–9.5 × 10^9^/L.
  - C-reactive protein <8 mg/L.
  - Procalcitonin <0.05 ng/mL.
6. Normal imaging and electrophysiology: No significant abnormalities on brain MRI/CT, EEG, or chest CT.
7. Data completeness: Availability of complete clinical data, identical to the requirements for the autoimmune encephalitis group.

Exclusion criteria (controls were excluded if they met any of the following):

1. History of neurodegenerative or neuroinflammatory diseases (e.g., multiple sclerosis, neuromyelitis optica spectrum disorders, CNS vasculitis, neurosarcoidosis, Parkinson’s disease, Alzheimer’s disease, spinocerebellar ataxia).
2. History of cerebrovascular disease, cranial or spinal structural injury, epilepsy, or status epilepticus.
3. History of confirmed or suspected central nervous system infection.
4. History of confirmed or suspected Guillain-Barré syndrome.
5. Diagnosis of systemic autoimmune disease (e.g., systemic lupus erythematosus, Sjögren’s syndrome, rheumatoid arthritis).
6. Active systemic infection or fever (axillary temperature >37.3 °C) within 4 weeks prior to enrollment.
7. History of intracranial tumors, leptomeningeal carcinomatosis, paraneoplastic syndromes, or other malignancies.
8. Immunodeficiency disorders or long-term use of immunosuppressants/corticosteroids.
9. History of hepatitis virus (HAV, HBV, HCV, HDV, HEV), HIV, or syphilis infection.
10. Familial hereditary diseases.
11. Pre-existing diabetes mellitus or thyroid disease.
12. Pregnancy or lactation.
13. History of alcohol or drug abuse/addiction.
14. Comorbid chronic nasal, oral, or skin diseases.

Participant screening and data collection: Investigators retrospectively screened the electronic medical records of The Second Affiliated Hospital of Soochow University according to the predefined inclusion and exclusion criteria from January 2010 to June 2025. The diagnosis of AE was based primarily on a compatible clinical phenotype and the reasonable exclusion of alternative causes, with neurological autoantibody test results serving as supportive diagnostic evidence. Patients were not classified as having AE solely on the basis of neurological autoantibody positivity. All potentially eligible cases identified during the initial screening were independently reviewed by two investigators. Disagreements were resolved through discussion or consultation with a third investigator, and only cases for which consensus was reached were included. The following data were collected from all enrolled participants in both case and control groups: sex, age, clinical symptoms, physical signs, medical history, electroencephalography (EEG), and brain magnetic resonance imaging (MRI) findings. Laboratory data included peripheral blood cell counts (white blood cell, absolute lymphocyte, absolute neutrophil, and absolute monocyte counts), serum total protein and albumin levels, and cerebrospinal fluid parameters, including opening pressure, cell count, total protein, albumin, and quantitative immunoglobulin levels (IgA, IgG, and IgM).

### 2.3 CSF-IgA measurement

CSF-IgA concentrations were measured in the central laboratory of the Second Affiliated Hospital of Soochow University. Following lumbar puncture, 2 mL of CSF was centrifuged at 3,000 rpm for 10 min, and the resulting supernatant was analyzed. CSF-IgA concentrations were determined using an immunoturbidimetric assay on a Beckman Coulter IMMAGE 800 analyzer with the Beckman Coulter Low Concentration Immunoglobulin A Test Kit (Beckman Coulter, Inc., Brea, CA, United States). The laboratory reference interval for CSF-IgA was 0–2 mg/L. The laboratory implemented internal quality-control procedures and participated in external proficiency-testing programs throughout the study period to ensure the reliability and consistency of the measurements.

### 2.4 Statistical analysis

All statistical analyses were performed using R software (version 4.2.2). Normality was assessed using the Shapiro–Wilk test. Non-normally distributed continuous variables were presented as medians with interquartile ranges and compared using the Mann–Whitney U test, while categorical variables were summarized as *n* (%) and compared using the chi-square test. Univariable logistic regression was used to screen variables associated with autoimmune encephalitis, and clinically relevant or statistically significant variables were entered into a multivariable logistic regression model. The final prediction model was selected based on statistical significance, model stability, clinical interpretability, and parsimony. Model discrimination was assessed using receiver operating characteristic curve analysis and the area under the curve with 95% confidence intervals. Calibration was evaluated using calibration plots, and clinical utility was assessed using decision curve analysis. For CSF-IgA, the cutoff was selected by maximizing the Youden index, and the sensitivity, and specificity were reported. A two-sided *p* value <0.05 was considered statistically significant.

## 3 Results

**Figure 1:**
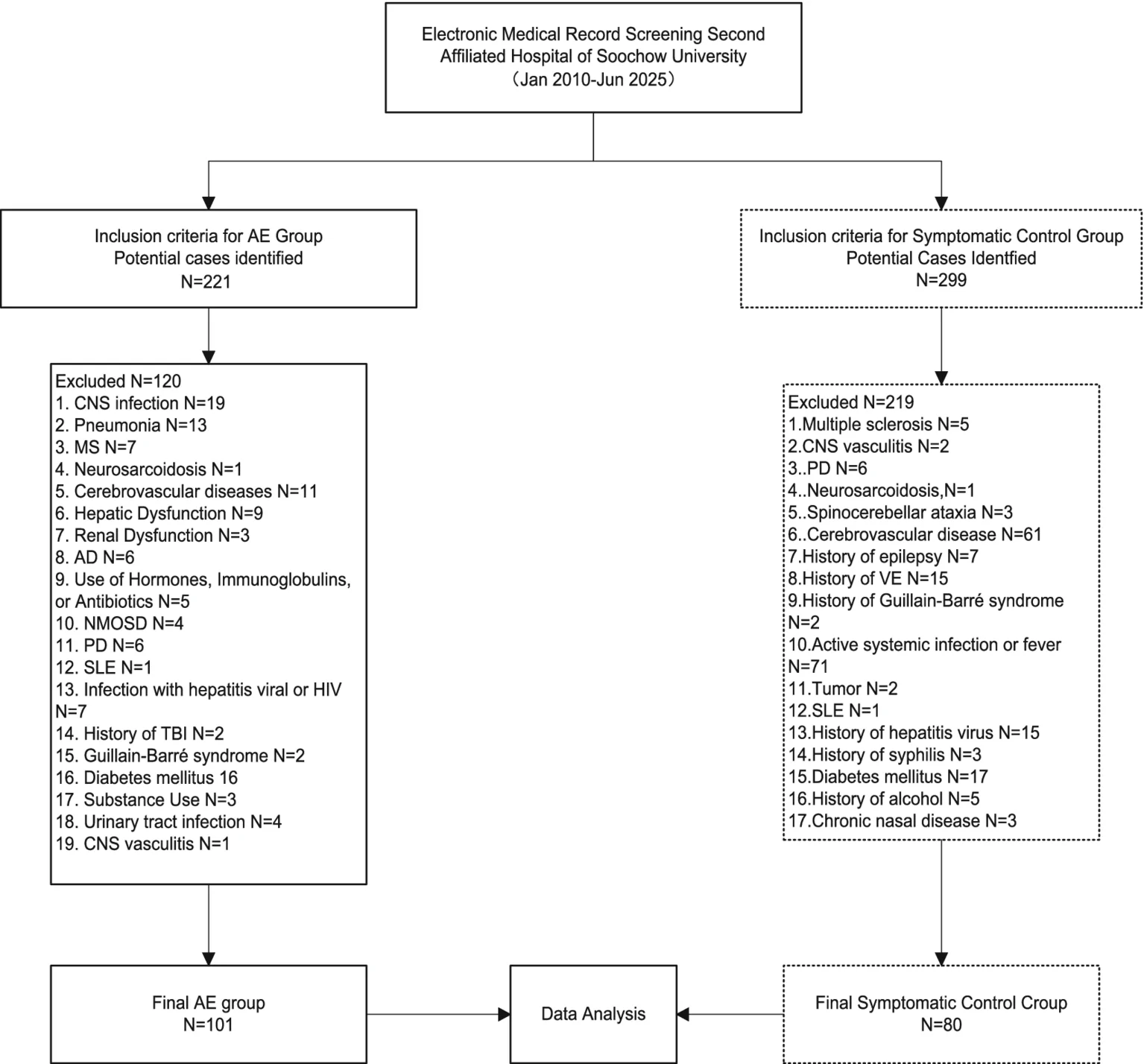
Flowchart of patient screening and cohort selection. AD, Alzheimer’s disease; MS, multiple sclerosis; NMOSD, neuromyelitis optica spectrum disorder; PD, Parkinson’s disorder; SLE, systematic lupus erythematosus; TBI, traumatic brain injury; CNS, central nervous system.

We initially identified 221 patients meeting the preliminary inclusion criteria for AE through a systematic screening of electronic medical records at the Second Affiliated Hospital of Soochow University between January 1, 2010, and June 30, 2025. After applying all pre-specified exclusion criteria, 120 patients were excluded, resulting in a final study cohort of 101 confirmed AE cases. Using the same screening procedures, 80 symptom control subjects were selected from the same source population and matched to the cases based on age and sex. The complete screening and enrollment process is detailed in Figure 1.

### 3.1 Baseline characteristics of the study population

This study included a total of 181 participants, comprising 101 patients in the AE group and 80 individuals in the symptomatic control group. The AE group consisted of 52 males and 49 females, with a median age of 52 years (IQR: 33.0–68.0). The control group included 39 males and 41 females, with a median age of 37.5 years (IQR: 32.0–54.0). Among the 101 AE patients, 57 exhibited abnormal electroencephalography (EEG) findings, characterized by diffuse or focal medium-to-high amplitude theta and delta waves, sharp waves or spikes, and sharp-spike-slow wave complexes. Thirty-six patients had normal EEG findings, whereas eight did not undergo EEG examination. Among the 37 patients, brain MRI revealed abnormal signal intensities suggestive of encephalitis, involving the frontal lobe, temporal lobe, insula, hippocampus, parietal lobe, brainstem, and cerebellum. No imaging abnormalities were observed in the remaining 64 patients. The antibodies detected in the cerebrospinal fluid of 101 patients included NMDAR (15), GFAP (21), AMPA (2), GABA (8), LG1 (5), mGluR5 (2), MOG (18), GAD65 (2), Yo (1), zic4 (1), Caspr2 (1), VGkc (1), Iglon5 (1), Gm2 (1) and CSF tissue-based assay positive (22).

Among the 101 patients with AE, 44 (43.6%) had a normal CSF WBC count, 33 (32.7%) had CSF total protein within the institutional reference range, and 16 (15.8%) had both indices within the normal range. Elevated total CSF IgA was observed in 42/44 (95.5%), 28/33 (84.9%), and 14/16 (87.5%) patients, respectively. Detailed data are shown in Supplementary Table S1. A descriptive summary of demographic characteristics and total CSF IgA levels stratified by antibody type and tissue-based assay (TBA) results is provided in Supplementary Table S2. Median CSF IgA levels were 3.22 mg/L in NMDAR encephalitis (*n* = 15) and 14.20 mg/L in GFAP astrocytopathy (*n* = 21). CSF IgA levels exceeded 2 mg/L in 80.0% (12/15) of NMDAR cases and in all GFAP, MOG, LGI1, and TBA-positive cases. Only subgroups with more than five patients are presented, and no formal subgroup comparisons were performed.

Comparison of baseline characteristics between the two groups revealed no significant differences in age or sex (*p* > 0.05). In contrast, the AE group showed significantly higher values in multiple CSF parameters (*p* < 0.05). Specifically, CSF levels of IgA, IgG, IgM, total protein, albumin, CSF WBC, blood WBC, and absolute neutrophil count were all markedly elevated in the AE group (all *p* < 0.001). Additionally, serum albumin levels were significantly lower in the AE group (*p* < 0.001). Detailed results are presented in Table 1.

**Table 1:** Baseline characteristics of the study population.

Variables	Control group (*n* = 80)	AE (*n* = 101)	*p*
Sex, *n* (%)			0.829
Female	41 (51.30)	49 (48.50)	
Male	39 (48.80)	52 (51.50)	
Age, years (Q1, Q3)	37.5 (32.00, 54.00)	52 (33.00, 68.00)	0.055
CSF parameters			
CSF-IgA, mg/L (Q1, Q3)	1.46 (1.24, 1.75)	6.03 (3.70, 12.30)	<0.001
CSF-IgG, mg/L (Q1, Q3)	22.5 (17.88, 28.50)	58.4 (33.60, 93.70)	<0.001
CSF-IgM, mg/L (Q1, Q3)	0.36 (0.23, 0.58)	1.32 (0.72, 2.69)	<0.001
CSF-TP, mg/L (Q1, Q3)	321 (253.00, 361.00)	492 (397.00, 836.00)	<0.001
CSF-Alb, mg/L (Q1, Q3)	174.15 (129.98, 225.00)	282.6 (191.20, 463.80)	<0.001
CSFWBC, ×10^6^/L	1 (0, 2.25)	9 (2.00, 45.00)	<0.001
ICP, mmH₂O	145 (120, 160)	140 (110, 190)	0.506
Blood parameters			
Serum Alb, g/L	44.1 (42.58, 46.05)	42 (38.10, 44.50)	<0.001
Serum Glob, g/L	23.8 (21.90, 27.12)	25.9 (23.40, 28.70)	0.007
WBC, ×10^9^/L	5.57 (4.80, 6.75)	7.3 (6.00, 9.50)	<0.001
ALC, ×10^9^/L	1.8 (1.40, 2.20)	1.6 (1.10, 2.10)	0.026
ANC, ×10^9^/L	3.4 (3.05, 4.73)	5.2 (3.70, 7.40)	<0.001
AMC, ×10^9^/L	0.4 (0.30, 0.50)	0.46 (0.38, 0.60)	0.009

Data are presented as *n* (%) or median (interquartile range). Alb, albumin; ALC, absolute lymphocyte count; AMC, absolute monocyte count; ANC, absolute neutrophil count; CSF, cerebrospinal fluid; Glob, globulin; Ig, immunoglobulin; TP, total protein; WBC, white blood cell count; ICP, intracranial pressure.

### 3.2 Univariable and multivariable logistic regression analyses

Univariable logistic regression analyses identified 11 candidate predictors significantly associated with AE (*p* < 0.05): age, CSF total IgA, CSF IgG, CSF IgM, CSF total protein, CSF albumin, CSF white blood cell (WBC) count, peripheral blood WBC count, peripheral blood absolute neutrophil count, serum albumin, and serum globulin. Older age; higher levels of CSF total IgA, CSF IgG, CSF IgM, CSF total protein, CSF albumin, and serum globulin; and higher CSF WBC, peripheral blood WBC, and peripheral blood absolute neutrophil counts were associated with increased odds of AE, whereas a higher serum albumin level was associated with decreased odds of AE.

These 11 candidate predictors were simultaneously entered into the initial full multivariable logistic regression model. Collinearity diagnostics of the initial model showed substantial multicollinearity, with particularly high VIFs for CSF total protein, CSF albumin, peripheral blood WBC count, and peripheral blood absolute neutrophil count (detailed data are shown in Supplementary Table S3.1). After mutual adjustment, six variables were statistically significant in the initial full model: CSF total IgA (adjusted OR = 31.63, 95% CI: 2.13–469.09, *p* = 0.011), CSF total protein (adjusted OR = 1.05, 95% CI: 1.01–1.10, *p* = 0.027), CSF WBC count (adjusted OR = 2.36, 95% CI: 1.08–5.16, *p* = 0.030), peripheral blood WBC count (adjusted OR = 6.50, 95% CI: 1.23–34.36, *p* = 0.027), CSF albumin (adjusted OR = 0.91, 95% CI: 0.84–0.97, *p* = 0.008), and serum globulin (adjusted OR = 0.68, 95% CI: 0.48–0.95, *p* = 0.023). Age, CSF IgG, CSF IgM, serum albumin, and peripheral blood absolute neutrophil count were not statistically significant in the initial full model. In the reduced six-variable model, VIF values ranged from 1.416 to 6.535, indicating no severe multicollinearity. Full regression results are presented in Table 2, and detailed collinearity diagnostics are shown in Supplementary Table S3.2.

**Table 2:** Univariable and multivariable logistic regression analyses of factors associated with autoimmune encephalitis.

Variable	OR [95% CI]	*p*-value	OR [95% CI]	*p*-value
Sex (male)	1.12 [0.62, 2.01]	0.715		
Age	1.03 [1.01, 1.04]	0.004	1.01 [0.95, 1.07]	0.752
CSF-IgA	11.53 [4.59, 29.01]	<0.001	31.63 [2.13, 469.09]	0.011
CSF-IgG	1.14 [1.09, 1.20]	<0.001	1.35 [0.99, 1.84]	0.058
CSF-IgM	16.68 [5.91, 47.08]	<0.001	0.60 [0.01, 39.44]	0.810
CSF-TP	1.01 [1.01, 1.02]	<0.001	1.05 [1.01, 1.10]	0.027
CSF-Alb	1.01 [1.01, 1.01]	<0.001	0.91 [0.84, 0.97]	0.008
CSF-WBC	1.47 [1.24, 1.75]	<0.001	2.36 [1.08, 5.16]	0.030
ICP	1.01 [1.00, 1.01]	0.126		
Blood parameters				
Serum-Alb	0.84 [0.77, 0.91]	<0.001	0.85 [0.62, 1.17]	0.319
Serum-Glob	1.10 [1.02, 1.18]	0.014	0.68 [0.48, 0.95]	0.023
WBC	1.38 [1.19, 1.60]	<0.001	6.50 [1.23, 34.36]	0.027
ALC	0.89 [0.64, 1.22]	0.467		
ANC	1.37 [1.17, 1.61]	<0.001	0.24 [0.06, 1.02]	0.051

### 3.3 Predictive model development and evaluation

Eleven predictors on 101 cases is about nine events per variable, at or below the usual floor, and an over-specified model with several correlated CSF measures gives an unstable estimate for any of them. Reducing the model cuts that instability, and the narrower interval is the more trustworthy of the two. Based on the results of the initial full multivariable analysis, we developed a reduced prediction model by retaining the six variables that were statistically significant in both the univariable analyses and the initial full multivariable model. The final reduced model included CSF total IgA, CSF WBC count, CSF total protein, CSF albumin, peripheral blood WBC count, and serum globulin. All six predictors were jointly refitted in the final model.

**Figure 2:**
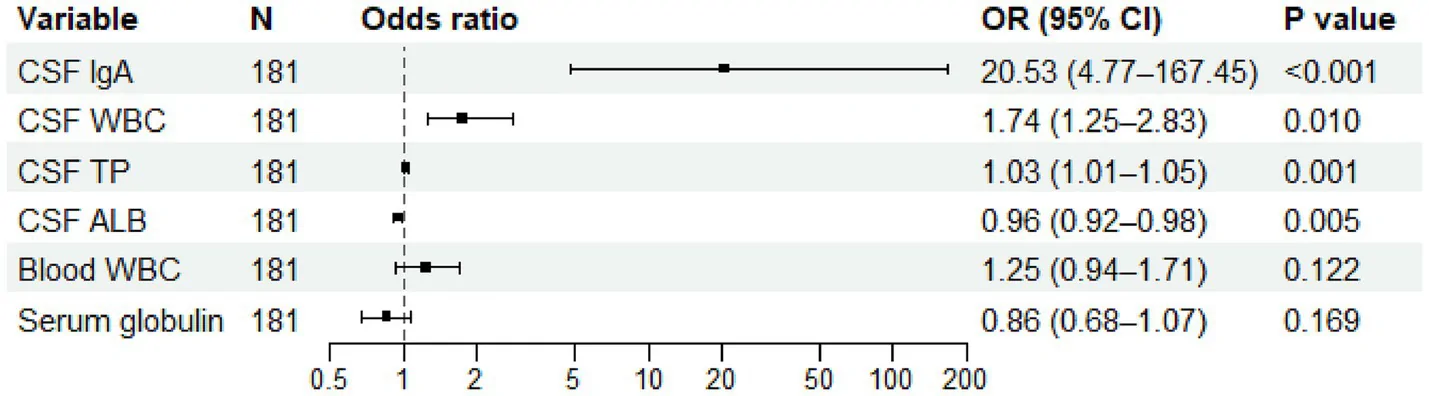
Forest plot of adjusted odds ratios and 95% confidence intervals for factors associated with autoimmune encephalitis in the multivariable logistic regression model.

The adjusted effect estimates in the final reduced model were as follows: CSF total IgA (adjusted OR = 20.53, 95% CI: 4.77–167.45), CSF WBC count (adjusted OR = 1.74, 95% CI: 1.25–2.83), CSF total protein (adjusted OR = 1.03, 95% CI: 1.01–1.05), CSF albumin (adjusted OR = 0.96, 95% CI: 0.92–0.98), peripheral blood WBC count (adjusted OR = 1.25, 95% CI: 0.94–1.71), and serum globulin (adjusted OR = 0.86, 95% CI: 0.68–1.07). The results of the final model are shown in Figure 2. Given the potential heterogeneity in the underlying pathophysiological mechanisms across AE subtypes, we performed a sensitivity analysis excluding patients with GFAP-, MOG-, Yo-, and Zic4-positive disease, which are not typically classified as AE proper. This exclusion removed 41 of 101 patients (approximately 40%) from the AE group, leaving 60 patients with AE and 80 controls for analysis. As shown in Supplementary Table S4, CSF total IgA remained independently associated with AE (OR 16.51, 95% CI 3.62–166.21), although the confidence interval was wider than that in the main model, and the main findings remained unchanged.

**Figure 3:**
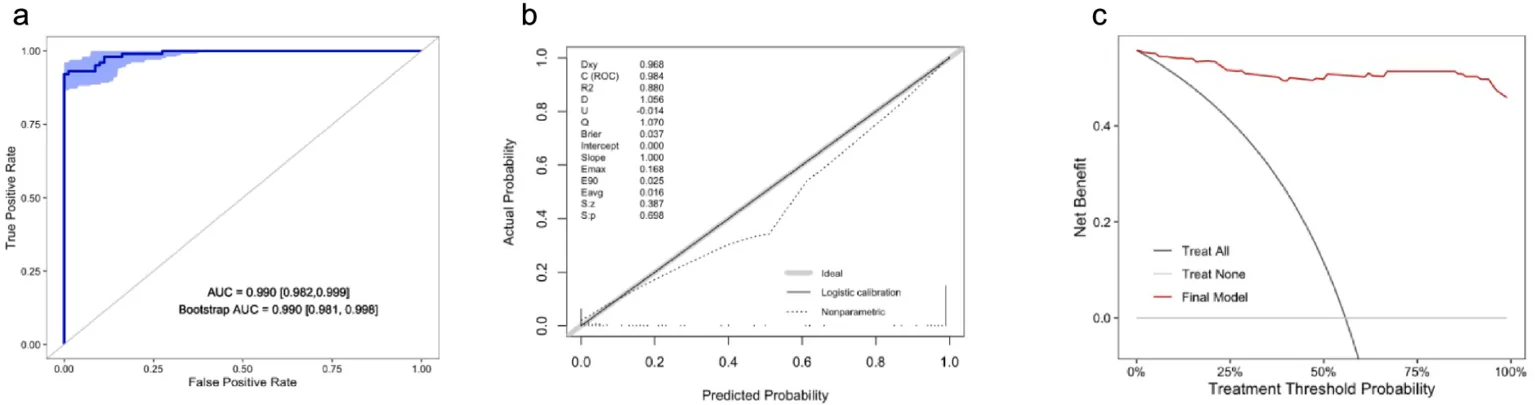
Performance of the multivariable model for identifying autoimmune encephalitis. **(a)** Receiver operating characteristic (ROC) curve of the prediction model. The model demonstrates strong discriminative ability in this validation subset, with an area under the curve (AUC) of 0.990 (95% CI: 0.982–0.999). Internal validation via bootstrap resampling (*n* = 2000) confirmed the robustness of this estimate (bootstrap AUC = 0.990, 95% CI: 0.981–0.998). **(b)** Calibration plot of the prediction model. The solid line represents the logistic calibration of the model, and the dashed line represents the ideal reference. Key calibration metrics include a Brier score of 0.037, a calibration slope of 1,000, and an intercept of 0.000. The maximum estimation error (Emax) was 0.168 and the average absolute error (Eavg) was 0.016, demonstrate a strong agreement between predicted probabilities and observed outcomes. **(c)** Decision curve analysis of the prediction model. The model demonstrated superior or non-inferior net benefit compared with Treat All/Treat None strategies across threshold probabilities of 0%–90%, with the greatest benefit at the 75% threshold, suggesting optimal clinical utility when high diagnostic certainty is required (e.g., before escalation to second-line immunotherapy).

The apparent performance of the final reduced model was assessed using the full cohort, and internal validation was performed using 2,000 bootstrap resamples to evaluate model stability and assess the extent to which its performance might have been overestimated due to overfitting. As shown in Figure 3, the model demonstrated good discrimination, calibration, and potential clinical utility. Receiver operating characteristic (ROC) curve analysis showed excellent apparent discrimination, with an area under the curve (AUC) of 0.990 (95% CI: 0.982–0.999; Figure 3a). Bootstrap internal validation yielded an AUC of 0.990 (95% CI: 0.981–0.998), indicating that the model’s discriminative performance was stable after internal validation.

The calibration plot showed close agreement between the predicted and observed probabilities of AE (Figure 3b). The model had a Brier score of 0.037, a calibration slope of 1.000, an Emax of 0.168, and an Eavg of 0.016. Decision curve analysis further showed that the model provided a greater net benefit than the treat-all and treat-none strategies across a range of clinically relevant threshold probabilities (Figure 3c). In addition, the diagnostic performance of total CSF IgA alone was evaluated. The optimal cutoff determined by maximizing the Youden index was 2.645 mg/L, yielding a sensitivity of 86.1% and a specificity of 98.8%, a sample-specific PPV of 98.9%, and a sample-specific NPV of 84.9% (Supplementary Figure S1).

## 4 Discussion

In this retrospective case–control study, we found that total CSF IgA was significantly associated with AE and remained independently associated with AE after adjustment for routine CSF and blood parameters. These findings suggest that total CSF IgA, a readily available laboratory parameter, may serve as a potentially useful adjunctive biomarker for early identification of AE.

IgA has been linked to several immune-mediated conditions involving the nervous system. Anti-NS1 IgA in plasma can enhance neutrophil activation in severe dengue (13), and MOG-IgA may serve as a potential diagnostic biomarker in patients with CNS demyelination (14). However, previous studies in AE have mainly focused on antigen-specific IgA antibodies, such as CSF IgA-type NMDAR antibodies (15), while the diagnostic relevance of total CSF IgA remains largely unexplored. Compared with antigen-specific antibody assays, total CSF IgA is easier to obtain in routine clinical practice and can be measured using standard laboratory platforms. It may therefore serve as a useful adjunctive biomarker marker, especially when comprehensive testing for anti-neural antibodies is unavailable or delayed.

The biological basis of elevated total CSF IgA in AE remains uncertain, and several non-mutually exclusive mechanisms may contribute. First, CNS immune activation may promote the recruitment or persistence of B cells and plasma cells and thereby support local immunoglobulin production (16, 17). Quantitative evidence of intrathecal IgA synthesis has been reported in a minority of patients with NMDAR encephalitis using paired serum–CSF measurements and Reiber-based analysis (18). Antigen-specific intrathecal IgA against synapsin has also been described in an individual patient with limbic encephalitis (19). These observations support the biological possibility of intrathecal IgA synthesis in selected patients but do not establish that it is common across AE subtypes or that it was the principal source of elevated total CSF IgA in our cohort.

Second, dysfunction of CNS barriers may increase the transfer of blood-derived immunoglobulins into the CSF. Biochemical evidence consistent with blood–brain barrier disruption, including alterations in CD44 and MMP9, has been reported in anti-NMDAR encephalitis (20). In addition, Lai et al. (21) found an elevated CSF/serum albumin quotient (QAlb) in 35.5% of patients with neuronal surface antibody-associated AE, indicating that blood–CSF barrier dysfunction occurs in a clinically relevant subset of patients. These findings support the biological plausibility that CNS barrier dysfunction may alter CSF protein and immunoglobulin composition in AE. However, neither study directly demonstrated increased transfer of circulating IgA into the CSF, and evidence from anti-NMDAR encephalitis or neuronal surface antibody-associated AE may not be generalizable to all AE subtypes.

Intrathecal synthesis and barrier-related transfer are not mutually exclusive and may coexist. The present retrospective study did not include calculation of the CSF/serum IgA quotient or Reiber-based estimation of intrathecal IgA synthesis. Therefore, our findings do not establish the source of CSF IgA or exclude a contribution from blood–CSF barrier dysfunction. Future prospective studies incorporating paired serum and CSF IgA measurements, QAlb assessment, and Reiber-based analysis of intrathecal IgA fractions are needed to clarify the origin of elevated CSF IgA in AE.

Third, more exploratory possibility is the trafficking of mucosal-educated IgA-producing cells to CNS-associated compartments. Gut-derived IgA-producing plasma cells have been identified in the meningeal compartment and in experimental neuroinflammation, while clonally related IgA-positive B cells have been detected in peripheral blood and CSF during multiple sclerosis activity (8, 11, 22, 23). These findings demonstrate that mucosal-educated IgA-producing cells can access CNS-associated compartments. However, comparable trafficking has not been directly demonstrated in AE, This mechanism should therefore be considered a hypothesis requiring direct investigation.

Overall, elevated total CSF IgA in AE may reflect intrathecal synthesis, increased blood-derived transfer across a dysfunctional blood–CSF barrier, immune-cell trafficking, or a combination of these processes. Our findings demonstrate an association but do not determine the source or functional role of CSF IgA. Moreover, because the present study excluded infectious and inflammatory neurological disorders from the control group, it cannot establish that CSF IgA is more prominently elevated in AE than in clinically relevant disease mimics. Elevated CSF IgA may therefore represent a broader neuroinflammatory response rather than an AE-specific process. Accordingly, the high discrimination observed in this selected case–control sample should not be interpreted as evidence of disease specificity or standalone diagnostic utility. Prospective studies should include infectious and inflammatory neurological controls, paired serum–CSF IgA measurements, age-adjusted albumin quotients, Reiber-based intrathecal fraction analysis, and longitudinal sampling.

From a clinical perspective, total CSF IgA may serve as a useful adjunctive biomarker because it is routinely available, relatively inexpensive, and generally faster to obtain than comprehensive neural autoantibody testing. It may provide additional information during the diagnostic evaluation of suspected AE, but should not be used as a standalone marker or as a substitute for established diagnostic criteria or neural autoantibody testing.

In the present cohort, an exploratory total CSF IgA cutoff of 2.645 mg/L yielded a sensitivity of 86.1%, a specificity of 98.8%, a PPV of 98.9%, and an NPV of 84.9% for distinguishing patients with AE from symptomatic controls. However, these predictive values are specific to the present study sample, in which the case proportion of 55.8% was determined by the case–control design and does not represent the prevalence or pre-test probability of AE in routine clinical practice. Although the use of symptomatic rather than healthy controls better reflects a differential diagnostic context, the predictive values remain dependent on the composition of the study cohort. In particular, the high PPV should not be directly generalized to clinical settings in which the pre-test probability of AE is substantially lower. Because this cutoff was derived and evaluated in the same single-center dataset, it should not be considered an established diagnostic threshold and requires external validation in an independent prospective cohort.

Descriptive subgroup analyses suggested that CSF IgA elevation was less consistent in NMDAR encephalitis, a common form of AE, than in the GFAP-, MOG-, and TBA-positive groups. This heterogeneity may have influenced the overall diagnostic performance. However, the small and uneven subgroup sizes preclude firm conclusions, and larger studies are needed to assess subtype-specific diagnostic performance.

Importantly, all AE cases in this study were positive for CSF neural autoantibodies. Therefore, these findings may not generalize to antibody-negative AE or unselected patients with suspected AE and unknown antibody status. The diagnostic and incremental value of total CSF IgA in these populations requires prospective, multicenter validation.

To further explore its potential for clinical integration, we constructed a multivariable model incorporating total CSF IgA and other routine laboratory parameters. Such a model may offer a practical approach to early risk stratification in patients with suspected AE, particularly in resource-limited settings or when neural antibody results are delayed. The model could complement existing diagnostic algorithms and help identify patients who may benefit from further evaluation. However, because it was developed in a single-center cohort, the model should be considered exploratory. Large-scale, prospective, multicenter studies are needed to externally validate the model and assess its discrimination, calibration, incremental value beyond existing diagnostic approaches, and clinical utility across diverse clinical settings and patient populations, including those with antibody-negative AE or unknown antibody status.

### 4.1 Limitations

Several limitations should be acknowledged. First, this was a single-center retrospective study, which may have introduced selection bias and limited the generalizability of the findings. Second, only neural autoantibody-positive cases were included to reduce potential diagnostic misclassification. Although this approach may have improved diagnostic specificity, it limits the generalizability of our findings to antibody-negative AE. The diagnosis of AE was based primarily on compatible clinical phenotypes and the reasonable exclusion of alternative etiologies, with neural autoantibody findings serving as supportive evidence. Nevertheless, the cohort included a subset of patients with compatible encephalitic phenotypes who tested positive for GFAP, MOG, Yo, or Zic4 antibodies and were classified within the broader AE spectrum, potentially introducing diagnostic and pathophysiological heterogeneity. Although the sensitivity analysis excluding atypical AE subtypes supported the robustness of our findings, residual heterogeneity across AE subtypes cannot be entirely excluded. Third, although the data permitted a descriptive examination of antibody subtypes, the small sample sizes within individual subtypes precluded formal subgroup comparisons and reliable subtype-specific conclusions. Larger studies with stratification by antibody type, clinical phenotype, and contemporary diagnostic and classification criteria are needed to clarify subtype-specific findings. Fourth, the symptomatic control group consisted mainly of patients with non-inflammatory neurological or functional neurological disorders. Although these patients were carefully selected as clinical comparators, their CSF profiles may differ from those of truly healthy individuals, potentially affecting estimates of the diagnostic performance of CSF IgA. Fifth, although total CSF IgA was independently associated with AE, the wide confidence interval indicates uncertainty in the effect size estimate, likely owing to the limited sample size and retrospective case–control design. Sixth, collinearity was observed in the initial full multivariable model, particularly among CSF total protein, CSF albumin, peripheral blood WBC count, and peripheral blood absolute neutrophil count. In the reduced final model, VIF values ranged from 1.416 to 6.535, indicating no severe multicollinearity, although the coefficient estimates for CSF total protein and CSF albumin should still be interpreted with caution because these variables are closely related (Supplementary Table S3.2). Finally, external validation and mechanistic investigations were not performed. Larger prospective multicenter studies are needed to externally validate and further refine our findings.

## 5 Conclusion

In this cohort, total CSF IgA was independently associated with AE, and a multivariable model incorporating CSF IgA and other routine laboratory parameters demonstrated excellent discrimination and good calibration in distinguishing patients with AE from symptomatic controls. As a readily available and rapidly measurable laboratory parameter, it may serve as an adjunctive biomarker for early identification of AE. Larger prospective, multicenter studies with independent external validation are needed to confirm the magnitude of the association, validate the diagnostic performance of total CSF IgA, and determine its clinical utility.

## Data Availability

The data analyzed in this study is subject to the following licenses/restrictions: the datasets are not publicly available due to institutional ethical restrictions and patient confidentiality. Access to the data is restricted to the research team and is permitted only for the purposes of this study. De-identified data may be made available from the corresponding author upon reasonable request, subject to approval from the Institutional Ethics Review Committee of The Second Affiliated Hospital of Soochow University. Requests to access these datasets should be directed to Corresponding author: Yongjun Cao, Prof. Email: yongjuncao@126.com.

## Glossary

AE: autoimmune encephalitis
ALC: absolute lymphocyte count
AMC: absolute monocyte count
AMPA: *α*-amino-3-hydroxy-5-methyl-4-isoxazolepropionic acid receptor
ANC: absolute neutrophil count
AUC: area under the curve
Caspr2: contactin-associated protein-like 2
CI: confidence interval
CNS: central nervous system
CRP: C-reactive protein
CSF: cerebrospinal fluid
CT: computed tomography
EEG: electroencephalography
FT3: free triiodothyronine
FT4: free thyroxine
GABA: gamma-aminobutyric acid
GAD65: glutamic acid decarboxylase 65
GFAP: glial fibrillary acidic protein
Gm2: GM2 ganglioside
HAV: hepatitis A virus
HBV: hepatitis B virus
HCV: hepatitis C virus
HDV: hepatitis D virus
HEV: hepatitis E virus
HIV: human immunodeficiency virus
HHV: human herpesvirus
HSV: herpes simplex virus
ICP: intracranial pressure
Ig: immunoglobulin
IgA: immunoglobulin A
IgG: immunoglobulin G
IgM: immunoglobulin M
Iglon5: immunoglobulin LON family member 5
LG1: leucine-rich glioma-inactivated protein 1
mGluR5: metabotropic glutamate receptor 5
MOG: myelin oligodendrocyte glycoprotein
MRI: magnetic resonance imaging
NMDAR: N-methyl-D-aspartate receptor
OR: odds ratio
PCT: procalcitonin
ROC: receiver operating characteristic
TBA: tissue-based assay
TBI: traumatic brain injury
TP: total protein
TSH: thyroid-stimulating hormone
ULN: upper limit of normal
VGkc: voltage-gated potassium channel complex
VZV: varicella-zoster virus
WBC: white blood cell
Yo: anti-Yo antibody
zic4: Zinc finger protein 4
VIF: variance inflation factor
