# Supplementary material for: Total cerebrospinal fluid IgA as a candidate adjunctive biomarker associated with autoimmune encephalitis: a retrospective case–control study
Source: Front Neurol. 2026 Aug 27;17:1934933. doi: 10.3389/fneur.2026.1934933 (PMC13557976; doi:10.3389/fneur.2026.1934933)
Supplement: Supplementary file 1 [file Table_1.DOCX]

**Table S1. Frequency of elevated total IgA in CSF according to CSF WBC count and total protein status among patients with AE**

| **Subgroup** | **Patients in subgroup, n/N (%)** | **Elevated CSF IgA, n/N** | **Proportion with elevated CSF IgA, % (95% CI)** |
| --- | --- | --- | --- |
| Normal CSF WBC count (≤5 ×10⁶/L) | 44/101(43.6) | 42/44 | 95.5 (84.9–98.8) |
| Normal CSF total protein level (150–450 mg/L) | 33/101(32.7) | 28/33 | 84.9(69.1–93.4) |
| Normal CSF WBC count and total protein level | 16/101(15.8) | 14/16 | 87.5(63.9–96.5) |

Abbreviations: AE, autoimmune encephalitis; CI, confidence interval; CSF, cerebrospinal fluid; IgA, immunoglobulin A; WBC, white blood cell.

**Table S2. Distribution of demographic characteristics and CSF total IgA levels according to antibody category and TBA finding**s

| **Antibody category** | **n** | **Female, n (%)** | **Age, median (IQR), years** | **CSF total IgA, median (IQR), mg/L** | **Range, mg/L** | **CSF total IgA >2 mg/L, n (%)** |
| --- | --- | --- | --- | --- | --- | --- |
| NMDAR | 15 | 10 (66.7%) | 31.0 (25.0–35.5) | 3.22 (2.04–14.50) | 0.80–25.30 | 12 (80.0%) |
| GABAR | 8 | 3 (37.5%) | 53.0 (44.5–65.3) | 7.40 (4.90–8.90) | 1.61–14.80 | 7 (87.5%) |
| LGI1 | 5 | 1 (20.0%) | 65.0 (62.0–74.0) | 11.00 (3.51–11.70) | 2.08–21.20 | 5 (100%) |
| GFAP | 21 | 11 (52.4%) | 55.0 (36.0–59.0) | 14.20 (5.35–22.40) | 2.79–47.10 | 21 (100%) |
| MOG | 18 | 9 (50.0%) | 33.5 (27.3–58.3) | 4.48 (4.02–6.20) | 2.06–15.40 | 18 (100%) |
| TBA-positive | 22 | 10 (45.5%) | 59.5 (43.3–72.5) | 7.06 (5.62–13.48) | 2.78–52.50 | 22 (100%) |

**Abbreviations:** CSF, cerebrospinal fluid; GABAR, γ-aminobutyric acid receptor; GFAP, glial fibrillary acidic protein; IgA, immunoglobulin A; IQR, interquartile range; LGI1, leucine-rich glioma-inactivated 1; MOG, myelin oligodendrocyte glycoprotein; NMDAR, N-methyl-D-aspartate receptor; TBA, tissue-based assay.
**Footnote:** Table S2 includes 89 of the 101 patients; the remaining 12 belonged to subgroups with five or fewer cases and are therefore not presented separately.

**Table S3.1. Multicollinearity assessment of variables included in the initial full multivariable model**

| Variable | VIF |
| --- | --- |
| Age | 1.261 |
| CSF-IgA | 4.002 |
| CSF-IgG | 7.301 |
| CSF-IgM | 3.040 |
| CSF-TP | 23.221 |
| CSF-ALB | 23.116 |
| CSF-WBC | 3.490 |
| Serum-Alb | 2.659 |
| Serum-Glob | 3.881 |
| Blood-WBC | 35.749 |
| Blood-ANC | 28.964 |

**Abbreviations:** CSF, cerebrospinal fluid; IgA, immunoglobulin A; IgG, immunoglobulin G; IgM, immunoglobulin M; WBC, white blood cell count; TP, total protein; ALB, albumin; GLB, globulin; VIF, variance inflation factor.

**Table S3.2. Multicollinearity assessment of variables included in the final model**

| Variable | VIF |
| --- | --- |
| CSF-IgA | 2.483 |
| CSF-WBC | 1.540 |
| CSF-ALB | 6.535 |
| CSF-TP | 5.273 |
| Blood-WBC | 1.416 |
| Serum-GLB | 1.555 |

**Abbreviations:** CSF, cerebrospinal fluid; IgA, immunoglobulin A; WBC, white blood cell; TP, total protein; ALB, albumin; GLB, globulin; VIF, Variance Inflation Factor.

**Table S4. Multivariable logistic regression analysis after exclusion of GFAP-IgG-, MOG-IgG-, anti-Yo-, and anti-Zic4-positive cases**

| Variable | Adjusted OR [95% CI] | p-value |
| --- | --- | --- |
| CSF-IgA | 16.51 [3.62, 166.21] | 0.004 |
| CSF-WBC | 1.74 [1.21, 3.01] | 0.022 |
| CSF-ALB | 0.96 [0.92, 0.99] | 0.011 |
| CSF-TP | 1.02 [1.00, 1.05] | 0.021 |
| Serum-WBC | 1.24 [0.92, 1.71] | 0.166 |
| Serum-GLB | 0.91 [0.70, 1.13] | 0.405 |

Abbreviations: AE, autoimmune encephalitis; CI, confidence interval; CSF, cerebrospinal fluid; IgA, immunoglobulin A; OR, odds ratio; WBC, white blood cell; TP, total protein; ALB, albumin; GLB, globulin. ORs were adjusted for all variables included in the model.

**Figure S1. Receiver operating characteristic curve of CSF-IgA for distinguishing patients with autoimmune encephalitis from control group.**

Footnote:The exploratory data-derived cutoff of 2.645 mg/L, selected by maximizing the Youden index, yielded a sensitivity of 86.1% and a specificity of 98.8%.
